# Supplementary material for: Content-rich biological network constructed by mining PubMed abstracts
Source: BMC Bioinformatics. 2004 Oct 8;5:147. doi: 10.1186/1471-2105-5-147 (PMC528731; doi:10.1186/1471-2105-5-147)
Supplement: Additional File 2 — The original results of the above study (non-essential files are deleted to keep the file size under the limit set by BMC bioinformatics). [file 1471-2105-5-147-S2.bz2 › chilibotAdditionalFile2/dip40/346ID10903799E4422/html/IL2RB_IL15.html]

 


 **IL2RB** and **IL15** 
  
Found 142 abstracts in PubMed, retrieved 40.  
 

 What does Google say? 
 PDF only 
| .edu only 

---

**Interactive relationship** (e.g. stimulation, inhibition, etc)

**Stimulatory relationship**- These studies unequivocally show that amniotic fluid lacks IL 2 and IL 15  [ **IL15** ]  but can stimulate CTLL 2 cell proliferation via the IL 2 receptor  [ **IL2RB** ]  beta chain.  Ref: 10712671 Immunology, 2000
- The major subset of CD122  [ **IL2RB** ]  hi CD44 hi CD8 cells is heavily dependent on IL 15  [ **IL15** ]  by three different parameters, namely 1 bystander proliferation induced via IFN induced stimulation of the innate immune system, 2 normal background proliferation, and 3 T cell survival.  Ref: 12370255 J Exp Med, 2002
- Interleukin 15  [ **IL15** ]  IL 15  [ **IL15** ]  is a novel cytokine that stimulates lymphocyte proliferation and migration via a trimeric receptor sharing the ss and gamma signal transducing chains with the IL 2 receptor  [ **IL2RB** ] .  Ref: 11134140 J Clin Endocrinol Metab, 2000
**Neutral relationship**- Interleukin IL 2 and IL 15  [ **IL15** ]  share the IL 2 receptor  [ **IL2RB** ]  betagamma c subunits IL 2Rbetagamma c but have specific, unique alpha receptor subunits.  Ref: 12453470 Cytokine, 2002
- Interleukin IL 15  [ **IL15** ] , which uses IL 2 receptor  [ **IL2RB** ]  R beta and gamma chains for signal transduction, shares many of the biological activities of IL 2.  Ref: 10823416 Cancer Immunol Immunother, 2000
- IL 15  [ **IL15** ]  dependency is most extreme for the Ly49 subset of CD122  [ **IL2RB** ]  hi CD44 hi CD8 cells.  Ref: 12370255 J Exp Med, 2002
- The receptors for interleukin 2 IL 2 and interleukin 15  [ **IL15** ]  IL 15  [ **IL15** ]  in T cells share the IL 2R beta subunit CD122  [ **IL2RB** ]  and gamma C subunit but have private alpha subunits.  Ref: 12121224 Immunol Cell Biol, 2002
- In contrast to CD122  [ **IL2RB** ]  hi cells, the CD122  [ **IL2RB** ]  lo subset of CD44 hi CD8 cells is IL 15  [ **IL15** ]  independent.  Ref: 12370255 J Exp Med, 2002
- Anti CD122  [ **IL2RB** ]  antibodies which block signaling through IL 2 receptor  [ **IL2RB** ]  beta chain, and therefore neutralize the effects of IL 15  [ **IL15** ]  and IL 2, also abrogated this enhancement.  Ref: 10906173 J Virol, 2000
- The functional receptors for IL 2 and IL 15  [ **IL15** ]  consist of a private alpha chain, which defines the binding specificity for IL 2 or IL 15  [ **IL15** ] , and shared IL 2 receptor  [ **IL2RB** ]  beta and gamma chains.  Ref: 11135625 Nat Med, 2001

**Non-interactive relationship** (e.g. studied together, co-existance, homology, etc.)

- Preincubation of cells with the Src family kinase inhibitor, PP1, surprisingly increased the IL 2 and IL 15  [ **IL15** ]  induced tyrosine phosphorylation of Gab2, indicating that an Src family kinase member negatively regulates IL 2 receptor  [ **IL2RB** ]  signaling in MF T cells.  Ref: 11340297 Exp Clin Immunogenet, 2001
- By characterizing BM cells expressing IL 15  [ **IL15** ]  receptor components, we found marked heterogeneity within the IL 2 receptor  [ **IL2RB** ]  beta chain CD122  [ **IL2RB** ]  subset, which included cells uniquely committed to the NK lineage.  Ref: 11433387 Eur J Immunol, 2001
- likewise, being CD122  [ **IL2RB** ]  lo, CD44 hi CD4 cells are IL 15  [ **IL15** ]  independent.  Ref: 12370255 J Exp Med, 2002
- BACKGROUNDTo define whether immunosuppressive agents that block the interleukin IL 2 pathway could prevent activation induced cell death of activated T cells in the graft, we measured expression of IL 2, IL 2 receptor  [ **IL2RB** ]  alpha chain CD25, IL 15  [ **IL15** ] , Fas, and Fas ligand by real time reverse transcription polymerase chain reaction in cardiac allografts.METHODSWe characterized the phenotype of the infiltrating cells CD3, CD68, CD25 by immunohistochemistry.  Ref: 12777860 Transplantation, 2003
- The present study investigated whether an explanation for the conflicting reports on the interleukin 2 IL 2 status of amniotic fluid is due to the presence of IL 15  [ **IL15** ]  which shares biological activities with IL 2 and utilizes the IL 2 receptor  [ **IL2RB** ]  beta chain.  Ref: 10712671 Immunology, 2000
- IL 15  [ **IL15** ] , but not IL 4, also induced tyrosine phosphorylation of Gab2, suggesting that the IL 2 receptor  [ **IL2RB** ]  beta chain is important for IL 2 induced Gab2 phosphorylation.  Ref: 11340297 Exp Clin Immunogenet, 2001
- SDZ RAD blocks lymphocyte stimulation by other cytokines e.g., IL 15  [ **IL15** ]  that are not inhibited by anti IL 2 receptor  [ **IL2RB** ]  mAb.  Ref: 10708100 Transplantation, 2000
- Signaling by IL 4, IL 7, and IL 15  [ **IL15** ] , which use IL 2 receptor  [ **IL2RB** ]  components, also was inhibited, indicating a block in T cell responses similar to that seen in immunodeficient patients lacking the IL 2 receptor  [ **IL2RB** ]  gamma chain or Jak3.  Ref: 10920190 Proc Natl Acad Sci U S A, 2000
- Differential effects of IL 2 and IL 15  [ **IL15** ]  on expression of IL 2 receptor  [ **IL2RB** ]  alpha.  Ref: 11478799 Biochem Biophys Res Commun, 2001
- Genes that were up regulated in cells exposed to D39 relative to those exposed to PLN included genes encoding proteins such as mannose binding lectin 1, lysozyme, alpha 1 catenin, cadherin 17, caspases 4 and 6, macrophage inflammatory protein 1beta MIP 1beta, interleukin 8 IL 8, monocyte chemotactic protein 3 MCP 3, IL 2 receptor  [ **IL2RB** ]  beta IL 2Rbeta, IL 15  [ **IL15** ]  receptor alpha IL 15Ralpha, interferon receptor 2, and prostaglandin E synthase.  Ref: 12654830 Infect Immun, 2003
- These are all tissues that express IL 15  [ **IL15** ]  receptor alpha but not IL 2 receptor  [ **IL2RB** ]  alpha.  Ref: 10910071 Cancer Res, 2000
- The plasma levels of a panel of cytokines and cytokine associated molecules IL 1alpha, IL 2, IL 4, IL 6, IL 10, IL 12, IL 15  [ **IL15** ] , macrophage colony stimulating factor M CSF, interferon gamma IFN gamma, tumor necrosis factor alpha TNF alpha, soluble IL 2 receptor  [ **IL2RB** ]  sIL 2R, soluble tumor necrosis factor receptor I or II sTNFRI or II were assessed in 56 plasma samples of 13 pediatric patients undergoing hematopoietic stem cell transplantation HSCT, bone marrow in 12 and cord blood in one from unrelated donors.  Ref: 11551026 Bone Marrow Transplant, 2001
